# Transcriptome-wide association study identifies putative elicitors/suppressor of *Puccinia graminis* f. sp. *tritici* that modulate barley *rpg4*-mediated stem rust resistance

**DOI:** 10.1186/s12864-019-6369-7

**Published:** 2019-12-16

**Authors:** Roshan Sharma Poudel, Jonathan Richards, Subidhya Shrestha, Shyam Solanki, Robert Brueggeman

**Affiliations:** 1 Department of Plant Pathology, North Dakota State University, Fargo, ND USA; 2 Department of Plant Pathology and Crop Physiology, Louisiana State University, Baton Rouge, LA USA; 3 Department of Crop and Soil Sciences, Washington State University, Pullman, WA USA

**Keywords:** *Puccinia graminis* f. sp. *tritici*, *Hordeum vulgare*, RMRL, Aviruelnce, Virulence, Effectors, Transcriptomics

## Abstract

**Background:**

Stem rust is an economically important disease of wheat and barley. However, studies to gain insight into the molecular basis of these host-pathogen interactions have primarily focused on wheat because of its importance in human sustenance. This is the first extensive study utilizing a transcriptome-wide association mapping approach to identify candidate *Puccinia graminis* f. sp. *tritici* (*Pgt*) effectors/suppressors that elicit or suppress barley stem rust resistance genes. Here we focus on identifying *Pgt* elicitors that interact with the *rpg4-*mediated resistance locus (RMRL), the only effective source of *Pgt* race TTKSK resistance in barley.

**Results:**

Thirty-seven *Pgt* isolates showing differential responses on RMRL were genotyped using Restriction Site Associated DNA-Genotyping by Sequencing (RAD-GBS), identifying 24 diverse isolates that were used for transcript analysis during the infection process. *In planta* RNAseq was conducted with the 24 diverse isolates on the susceptible barley variety Harrington, 5 days post inoculation. The transcripts were mapped to the *Pgt* race SCCL reference genome identifying 114 K variants in predicted genes that would result in nonsynonymous amino acid substitutions. Transcriptome wide association analysis identified 33 variants across 28 genes that were associated with dominant RMRL virulence, thus, representing candidate suppressors of resistance. Comparative transcriptomics between the 9 RMRL virulent -vs- the 15 RMRL avirulent *Pgt* isolates identified 44 differentially expressed genes encoding candidate secreted effector proteins (CSEPs), among which 38 were expressed at lower levels in virulent isolates suggesting that they may represent RMRL avirulence genes. Barley transcript analysis after colonization with 9 RMRL virulent and 15 RMRL avirulent isolates inoculated on the susceptible line Harrington showed significantly lower expression of host biotic stress responses specific to RMRL virulent isolates suggesting virulent isolates harbor effectors that suppress resistance responses.

**Conclusions:**

This transcriptomic study provided novel findings that help fill knowledge gaps in the understanding of stem rust virulence/avirulence and host resistance in barley. The pathogen transcriptome analysis suggested RMRL virulence might depend on the lack of avirulence genes, but evidence from pathogen association mapping analysis and host transcriptional analysis also suggested the alternate hypothesis that RMRL virulence may be due to the presence of suppressors of defense responses.

## Introduction

Plants are subjected to a diverse array of microbes that can trigger a two-tiered immune response [1]). The first tier involves the recognition of conserved microbe-derived molecules known as pathogen-associated molecular patterns (PAMPs) or more appropriately as microbe-associated molecular patterns (MAMPs) by cell surface receptors. The MAMPs are essential for survival and conserved across diverse genera, thus cannot be shed by the pathogen and are under purifying selection. Well-characterized examples of MAMPs are bacterial flagellin, which is required for motility [2, 3] and the structural molecule chitin found in fungal cell walls [4]. The conserved bacterial flagellin subunit, flg22, is recognized by the host membrane-localized pattern recognition receptor (PRR) FLS2 [5], and the conserved fungal cell wall component chitin is recognized by another host PRR receptor, CERK1 [6, 7]. These broad classes of cell surface receptors known as receptor-like kinases (RLKs) typically trigger PAMP-triggered immunity (PTI) responses that are effective early defenses referred to as non-host resistances that confer resistance to a broad spectrum of pathogens [5, 8, 9]. For a microbe to become a specialized pathogen on a host it must overcome these PTI or non-host resistance responses, which requires the evolution of effectors that suppress PTI responses and manipulate host cell physiology to facilitate nutrient acquisition and ultimately reproduction. However, plants coevolved with specialized pathogens resulting in the second tier of defense responses that rely on race-specific resistance (R)-genes that recognize the action of these virulence effectors to elicit a higher amplitude of defense responses known as effector triggered immunity (ETI). Pathogen effectors or their action on the host are recognized by *R*-genes, triggering the ETI response effectively transforming the virulence effectors into avirulence (Avr) genes [10, 11]. Typically, ETI responses activate localized and pronounced programmed cell death known as the hypersensitive response (HR).

*Puccinia graminis* f. sp. *tritici* (*Pgt*) is an obligate biotrophic fungal pathogen that causes the economically important disease stem rust in wheat (*Triticum aestivum*) and barley (*Hordeum vulgare*) [12]. Obligate biotrophs proliferate on living host tissue by hijacking normal cellular physiological processes to facilitate the acquisition of nutrients to power their own growth and eventual sporulation [13]. During the infection process, *Pgt* develops an appressorium over the top of stomata and penetrates the host with an infection peg that breaches the guard cell barrier and allows for substomatal intercellular growth. Once the intracellular infection hyphae encounter mesophyll cells, they breach the cell wall, invaginate the host plasma membrane and form a specialized feeding structure called the haustorium [14, 15]. The haustorium act as the focal point of host-pathogen interaction through which fungal effectors that function to manipulate the host machinery are delivered into the host cytoplasm [15, 16]. Effectors are directly or indirectly recognized by cytoplasm localized R-proteins triggering resistance responses. Thus, the identification and characterization of virulence effectors is imperative for understanding and deploying durable resistances.

Transcriptomics/RNAseq has proven to be an instrumental molecular tool to help identify virulence effectors and avirulence genes as well as fill knowledge gaps in the understanding of the molecular mechanisms that determine the outcome of virulence effector manipulation, *R*-gene detection and the resulting compatible and incompatible interactions. As both the host and pathogen interact in this closely orchestrated a battle for supremacy, the underlying transcriptional regulation of gene expression in the plant and pathogen provide clues to their reactions and counter reactions [17–19].

In past research focused on characterizing cereal-rust pathosystems, RNAseq has been extensively used to characterize the transcriptional changes in both the host and pathogen at different stages of infection [17–22]. In addition, to utilizing the transcriptomics data to identify differentially regulated genes during compatible and incompatible interactions, the data can also be mined for genetic variation including single nucleotide polymorphism (SNPs) and/or insertion/deletions (INDELs). These polymorphic markers can be used to perform association analyses to identify marker-trait associations (MTA) with virulence/avirulence in the pathogen [23] [23]. conducted a transcriptome-wide association mapping study using data from 17 *Blumeria graminis* f. sp. *hordei* (*Bgh*) isolates and identified *AVR*_*a1*_ and *AVR*_*a13*_ as the avirulence effectors recognized by the *Mla1* and *Mla13* R-gene alleles, respectively, based on non-synonymous SNPs in the candidate effector genes identified.

The wheat stem rust resistance gene *Rpg1* is currently the only source of stem rust resistance deployed in the Upper Midwestern US and Canadian Prairies provinces, a major barley-growing region of North America [24]. Thus, barley production in these regions conducive to stem rust epidemics is vulnerable because of the emergence of the domestic *Pgt* race QCCJB [25, 26] and race TTKSK (aka Ug99) and its lineage in Africa [27], that are virulent on *Rpg1*. The only effective resistance to *Pgt* races QCCJB and TTKSK in barley is the *rpg4-*mediated resistance locus (RMRL) that required the concerted action of three tightly linked genes: two NBS-LRR (NLR) resistance-like genes, *Rpg5* and *HvRga1*, and the actin depolymerization factor *HvAdf3* that are required together for resistance [28–31].

Allele analysis from a diverse set of *Pgt* race QCCJB resistant lines carrying a functional RMRL and susceptible barley lines determined that *HvRga1* and *HvAdf3,* although required for resistance, are conserved genes with no functionally relevant polymorphism that explain RMRL function [31]. Despite the different recessive -vs- dominant nature of resistance between the wheat stem rust *R*-gene *rpg4* and the rye stem rust *R*-gene *Rpg5* it appeared that the functional polymorphism in *Rpg5,* primarily the STPK to protein phosphatase 2C domain insertion/deletion, showed that it is the polymorphic resistance gene responsible for *rpg4*-mediated stem rust resistance in barley [32].

Although the combination of the *Rpg1* and RMRL confers resistance to all currently characterized rust isolates/races, the presence of isolates/races that are virulent on *Rpg1* or RMRL just in the North Dakota (ND) *Pgt* population suggests that isolates with both virulences may exist or be generated, especially in sexual *Pgt* populations. The possibility of this combination of genes occurring in North America has been greatly diminished by stabilizing the *Pgt* population by the removal of the secondary host through the barberry eradication program [12]. However, continued surveillance of diverse *Pgt* races to detect virulence patterns on both barley stem rust resistance genes is important because this combination of virulence or lack of avirulence certainly could emerge in other regions of the globe where the sexual stage of the pathogen still occurs. It is also important to focus basic research efforts to understand the molecular mechanism underlying the broad *Rpg1* and RMRL resistance mechanisms to get a better evolutionary understanding of the barley-*Pgt* pathosystem. It appears that barley is a near non-host or recent host of *Pgt* as little co-evolution of race-specific resistances have evolved. Also, there is an interaction between *Rpg1* and RMRL resistance mechanisms and other loci within the barley genome that must be considered when pyramiding the genes in elite barley backgrounds to achieve broad stem rust resistance [33].

The majority of research including previous gene expression studies performed during cereal host-rust pathogen interactions were focused on hexaploid wheat [17–20, 34–36] due to its importance concerning world food security. Despite, barley being an economically important cereal crop worldwide and equally vulnerable to rust in the absence of effective stem rust resistance genes, no *in-planta* transcriptomic study had been conducted during barley-rust interactions to our knowledge.

In the present study, a total of 37 *Pgt* isolates were initially utilized to assay their virulence patterns on *Rpg1* and RMRL and to assess their diversity. Twenty-four diverse isolates were selected to conduct *in planta* transcriptomic analysis during the infection cycle on the susceptible barley variety Harrington. Since, previous studies reported a direct interaction between the avirulence effector of *M. lini* [37], and *Pgt* [36, 38] with their cognate *R*-genes, this study was initially aimed to identify avirulence effectors in *Pgt* isolates that are specifically recognized by *Rpg1* and *Rpg5*. The overall objectives of this study were to: a) identify *Pgt* isolates that are virulent/avirulent on *Rpg1* and RMRL, b) utilize *in planta* transcriptomics data to identify differentially expressed host and pathogen genes, and c) utilize *Pgt* gene expression data to conduct a transcriptome-wide association mapping to identify variants associated with virulence/avirulence specific to *Rpg1* and RMRL.

## Results

### Phenotypic assay

Based on the virulence pattern on the only effective wheat stem rust resistance genes in barley, *Rpg1* and RMRL, the *Pgt* isolates used in this study were placed into three groups*;* group 1 isolates were virulent on barley lines with RMRL only; group 2 isolates were virulent on barley lines with *Rpg1* only; and group 3 isolates were not virulent on barley carrying either *Rpg1* or RMRL. A fourth group, group 4 isolates would be virulent on barley lines containing both *Rpg1* and RMRL, however none of the isolates tested contained a distinct virulence pattern on both genes. Only four of the 37 isolates selected, R29JA and R29JB (group 1), QCC-2 (group 2) and A-5 (group 3) had been previously assayed for seedling resistance on barley lines containing these two differential resistance genes. Though R29JA and R29JB were both race typed as HKHJ, they were obtained from different sources and both isolates were included in this study. The phenotypic assays identified 9 group 1, 8 group 2 and 20 group 3 *Pgt* isolates (Additional file 1: Table S1-S4). Although the isolate A-15 did show a moderate level of aggressiveness/virulence on barley line Q21861 (*Rpg1+* and RMRL) and Chevron (*Rpg1+*), this isolate had a higher level of aggressiveness on HQ1 (RMRL) and Morex (*Rpg1+*), the discrepancies of virulence on the *Rpg1* carrying line Chevron and Morex and moderate virulence on Q21861 complicated the grouping of this isolate as group 4. Based on its consistent virulence on HQ1, we decided to place it under the group 3 isolates. Thus, no isolates clearly belonging to group 4 were identified in this study. The infection types of the *Pgt* isolates belonging to the three groups are shown in Additional file 1: Table S2-S4. For comparison in RNAseq and AM, 9 group 1 and 7 group 3 isolates were designated as avirulent on *Rpg1* (*AvrRpg1*) and 8 group 2 and 7 group 3 isolates were designated as avirulent on RMRL or *rpg4* (*Avrrpg4*) (Additional file 1: Table S1).

### Diversity assay using RAD-GBS to select isolates for RNAseq

**Fig. 1:**
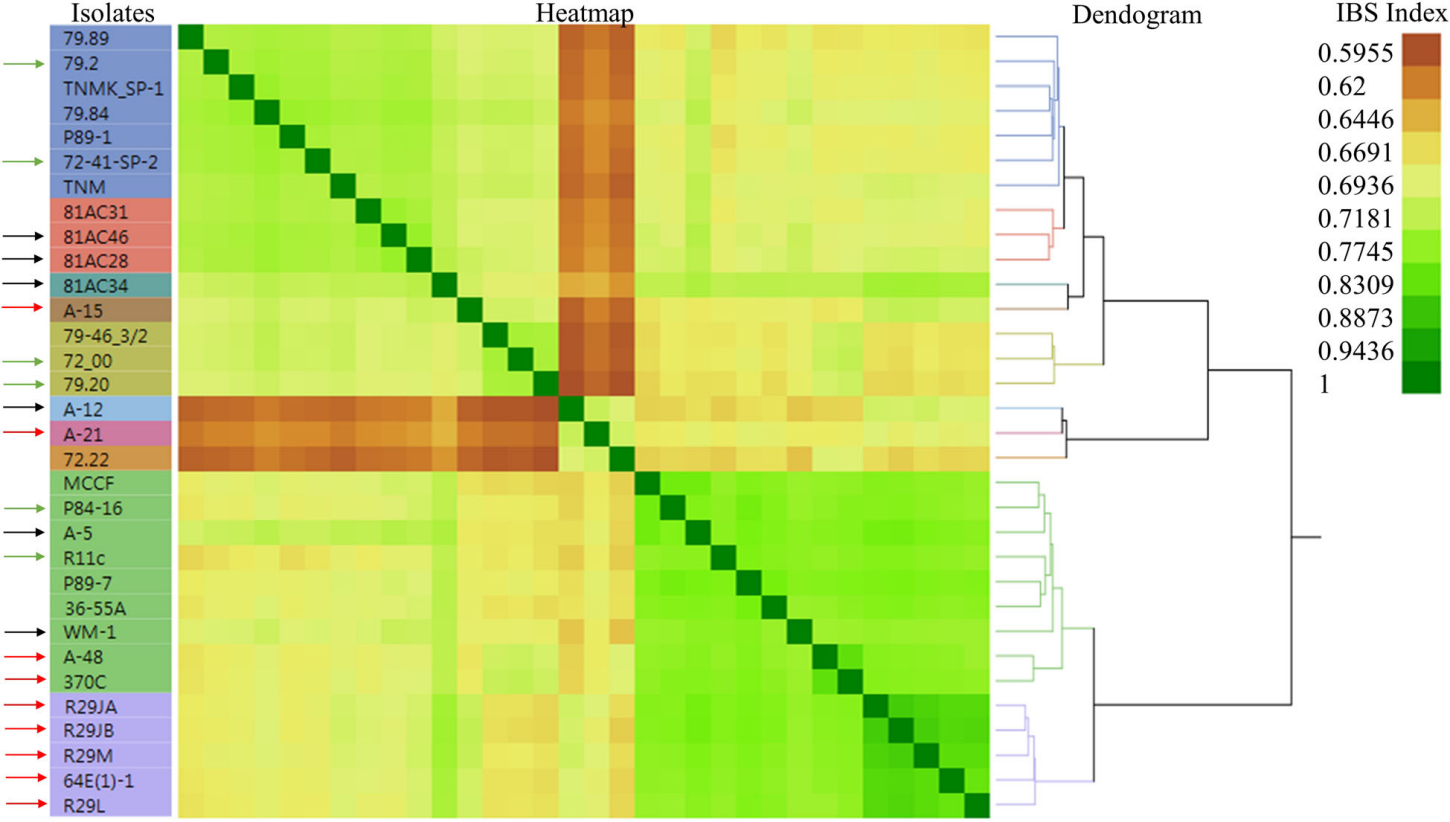
Heatmap and dendrogram of 32 *Pgt* isolates with differential virulence on barley lines with and without the stem rust resistance genes RMRL and/or *Rpg1*. Dendogram was obtained using hierarchical clustering (Fast Ward method) on the genotypic data generated for the 32 *Pgt* isolates used in this study. The colors of heatmap represents the relationship matrix [Identity by State (IBS)] value (shown on top right corner) that explain the degree of relatedness between the isolates. IBS with a value of 1 represents a perfect relationship and are dark red, while the decreasing IBS values represents increasing genetic diversity between two isolates compared. The isolates with an arrow on the left are the virulent RMRL (red arrows)*,* virulent *Rpg1* (green arrows) and avirulent RMRL and *Rpg1* (black arrows) *Pgt* isolates selected for RNAseq analysis based on this diversity assay

Sequencing data from 4 different size selected (200 bp, 240 bp, 275 bp, and 300 bp) RAD-GBS libraries were combined to obtain a single FASTQ file. Following concatenation, reads were mapped to *Pgt* strain CRL 75–36–700-3 (race SCCL; Accession: PRJNA18535) reference genome sequence obtained from the Broad Institute website [39, 40]. Five samples were removed from the analysis due to poor quality because of bad sequencing data and poor alignment. On average 545.9 K (S.D. ±290.5 K) reads were obtained from the remaining 32 samples (Additional file 1: Table S5). The percentage of reads aligned to the *Pgt* reference genome ranged from 55.48 to 87.28% with an average alignment of 78.70% (S.D. ±9.05%). Variant calls followed by several filtering parameters (details in the materials and methods) resulted in 11,423 markers for AM analysis. The AM did not result in a significant association between the different groups of *Pgt* and specific virulence on barley lines containing the RMRL or *Rpg1* resistance genes. However, a relation matrix constructed to obtain identity by state for running the Q-K model for AM was used to assess the diversity in group 3 *Pgt* isolates (Fig. 1). Seven group 3 *Pgt* isolates that were comparatively diverse compared to each other and other isolates in group 1 and group 2, were selected for the *in planta* RNAseq experiment.

### RNAseq reads alignment statistics

**Fig. 2:**
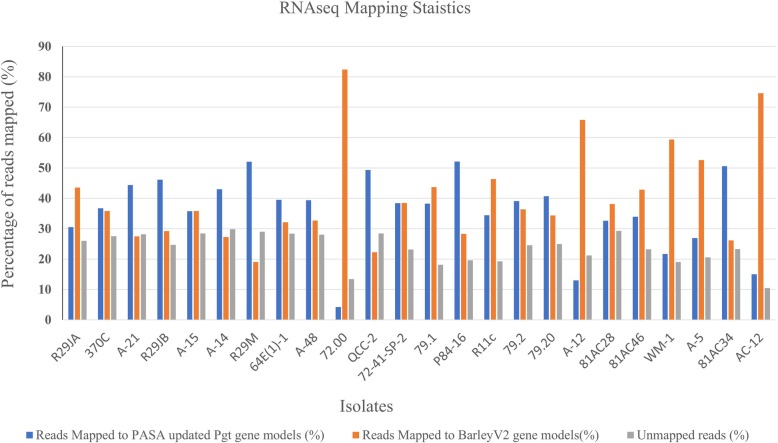
Bar graph showing the mapping statistics for the RNAseq reads on the *P. graminis* f. sp. *tritici* and barley reference gene models. The data represented in this bar graph is provided in Appendix Table S6

A total of 1.2 billion single end reads ranging from 34 million (M) to 82 M per sample were generated from two different runs on an Illumina NextSeq 500. After demultiplexing and quality trimming, a total of 1.12 billion reads yielding an average of 46.7 M (S.D. ± 14.4 M) reads per sample were generated (Additional file 1: Table S6). The average percentage of reads that mapped to the PASA updated *Pgt* SCCL gene models (Additional files 2 and 3) were 35.73% (S.D. ± 12.36%), among which 34.45% (S.D. ± 11.92%) uniquely mapped and 0.91% (S.D. ± 0.32%) mapped to multiple locations in the genome (Additional file 1: Table S6; Fig. 2).

Likewise, 40% (S.D. ± 16.12%) of the reads mapped to the barley RefSeq v1.0 gene models (Additional file 1: Table S6; Fig. 2). On average, 34.33% (S.D. ± 13.73%) of the total mapped reads aligned to a single unique locus in the barley genome and 6.27% (S.D. ± 2.43%) aligned to multiple positions (Additional file 1: Table S6). After alignment to the *Pgt* and barley reference sequences, 23.67% (S.D. ± 5.05%) of the sequencing reads were extracted as unmapped. In the non-inoculated control samples, 46.4 M (S.D. ± 2.9 M) reads were obtained from three replicates (Additional file 1: Table S7). On average, 81.94% (S.D. ± 1.24%) of the reads mapped to the barley genome, of which 64.71% S.D. ± 0.65%) mapped to unique positions in the genome. Approximately 35.29% (S.D. ± 0.65%) did not map to the barley reference genome sequence.

### Differential expression of host-specific genes in response to *Pgt* with varying virulence profile

To test the hypothesis that the difference in the virulence profile of *Pgt* alters the host-specific expression of genes involved in immune responses, we performed a comparative assay of host-specific gene expression between samples inoculated with *Pgt* isolates showing differential virulence profiles. The comparison between samples inoculated with RMRL virulent vs avirulent isolates using the host expression data set identified 115 high confidence barley genes that were differentially expressed (Additional file 1: Table S8). Fifty-seven of these DEGs exhibited a significantly lower expression pattern in samples inoculated with RMRL virulent *Pgt* isolate, 10 of which were predicted to encode different classes of heat shock proteins that are known for their role in plant defense response (Table 1; Additional file 1: Table S8 [41, 42];). To confirm if these lower levels of expression are due to the suppression by RMRL virulent isolates, host-specific expression of genes were compared between *Pgt* inoculated and non-inoculated controls. This later analysis showed that these 57 genes (Additional file 1: Table S8) were not significantly different in RMRL-virulent inoculated samples compared to the non-inoculated control (Table 1; Additional file 1: Table S8). In contrast, they were expressed significantly higher in RMRL-avirulent inoculated samples when compared to the non-inoculated controls (Table 1; Additional file 1: Table S8), thus confirming that the RMRL-virulent isolates suppress these host-specific genes.

**Table 1:** List of heat shock proteins have suppressed expression in samples inoculated with virulent *rpg4/5* isolates when compared to avirulent *rpg4/5*

Gene ID	Barley Annotation	Vir *rpg4/5* vs Avr *rpg4/5*		Vir *rpg4/5* inoculated vs Control		Avr *rpg4/5* inoculated vs Control	
		FC^a^	P-value^b^	FC^c^	P-value^b^	FC^d^	*P*-value^b^
HORVU4Hr1G063350	heatshockprotein21	−24.9	3.6E-07	3.8	0.88	94.9	5.57E-04
HORVU3Hr1G007500	16.9kDaclassIheatshockprotein1	−14.5	3.6E-09	20.4	0.05	307.4	4.95E-07
HORVU2Hr1G077710	22kDaclassIVheatshockprotein	−11.9	4.6E-03	2.3	1.00	27.9	9.51E-02
HORVU4Hr1G060760	17.9kDaclassIheatshockprotein	−10.4	2.4E-04	10.8	0.77	118.5	1.62E-03
HORVU3Hr1G006530	16.9kDaclassIheatshockprotein1	−8.8	1.6E-06	6.4	1.00	59.7	1.18E-03
HORVU4Hr1G059260	Heatshock70kDaprotein3	−6.1	1.2E-03	14.1	0.04	85.5	8.02E-05
HORVU4Hr1G067430	Heatshock70kDaprotein8	−5.2	1.5E-02	4.4	1.00	23.5	1.94E-01
HORVU4Hr1G015170	17.6kDaclassIIheatshockprotein	−4.2	4.4E-02	10.9	0.77	47.5	1.23E-02
HORVU3Hr1G007380	16.9kDaclassIheatshockprotein2	−3.9	6.4E-04	21.1	0.12	87.23	6.09E-05
HORVU7Hr1G081510	DnaJ/Hsp40cysteine-richdomainsuperfamilyprotein	−3.63	2.0E-06	−22.69	0.00	−6.33	0

^a^Negative values represents significantly lower expression of genes specific to *rpg4/5*-virulent *Pgt* inoculated samples compared to *rpg4/5* avirulent *Pgt* inoculated samples

^b^False discovery rate (FDR) corrected P-value. FDR corrected P-value < 0.05 are considered as significantly differentially expressed for given comparison

^c^Negative values represents significantly lower expression of genes specific to *rpg4/5*-virulent *Pgt* inoculated samples compared to non-inoculated control samples

^d^Positive values represents significantly higher expression of genes specific to *rpg4/5*-avirulent *Pgt* inoculated samples compared to non-inoculated controls

**Fig. 3:**
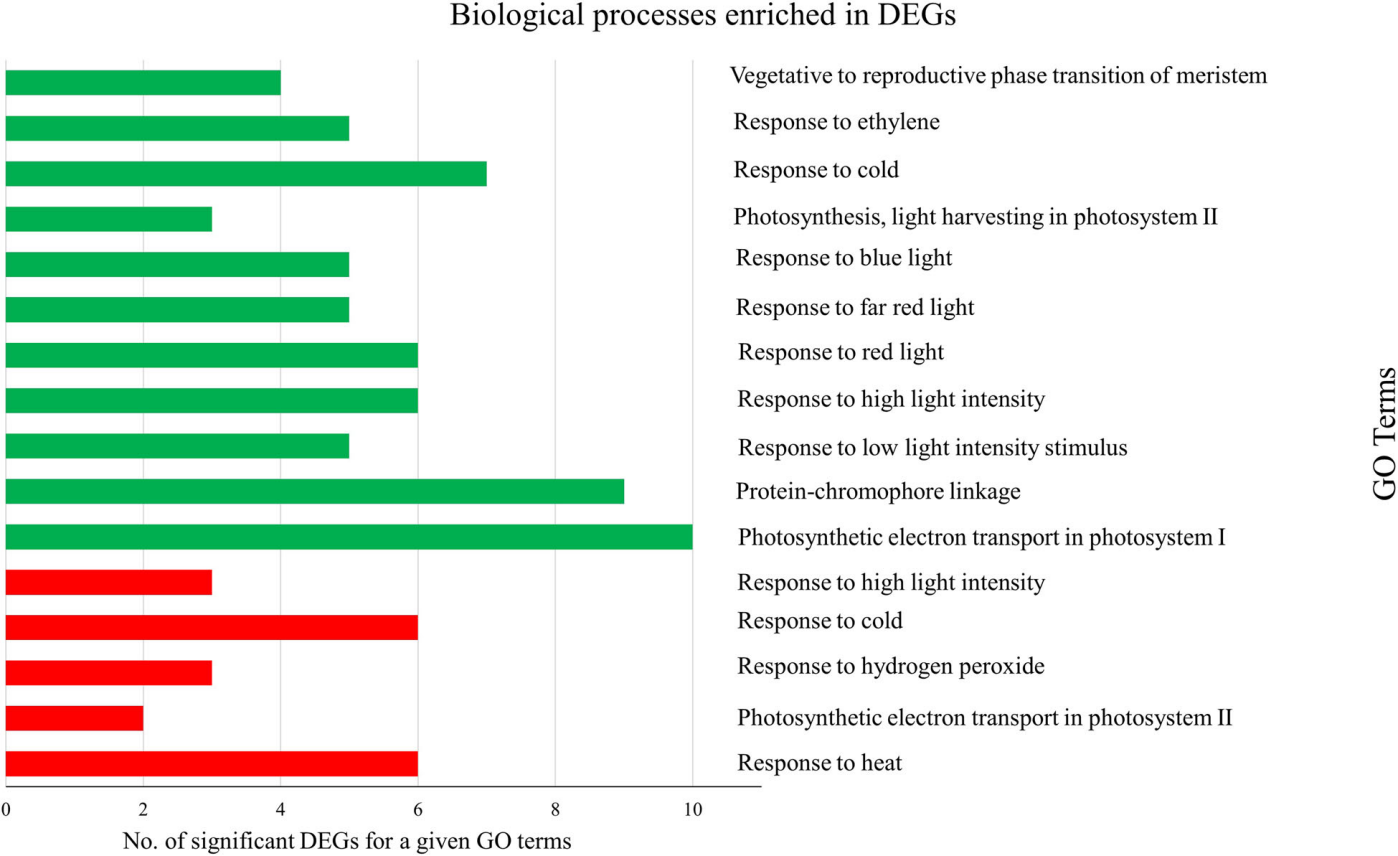
Bar graph showing the number of differentially expressed barley genes identified utilizing gene ontology (GO) enrichment analysis for involvement in specific biological processes. Red color represents genes that are expressed significantly higher and green color represents genes that are expressed significantly lower level in samples inoculated with RMRL virulent *Pgt* isolates when compared to RMRL avirulent *Pgt* isolates

Gene enrichment analysis were conducted using two groups of genes, those expressed at significantly higher and lower levels in samples inoculated with RMRL virulent *Pgt* isolates compared to samples inoculated with RMRL avirulent *Pgt* isolates. For each set of DEGs, GO terms that were significantly enriched for subontology biological process, molecular function and cellular component were identified. The low expressed genes were enriched for response to temperature, electron transport of photosystem II, and light intensity (Additional file 1: Table S9; Fig. 3). All of the genes encodings sHSP and HSPs (Table 1) were annotated to a GO term GO:0009408 associated with ‘response to heat’. The highly expressed genes were enriched for reaction to different light spectrum and light intensity, components of photosystems I and II, and responses to ethylene and cold stress. Enrichment for cellular components suggested that the majority of the DEGs act or are involved in chloroplast activity (Additional file 1: Table S9). Only the genes exhibiting a higher expression in this comparison were enriched for GO term subontology MF (Molecular Function). Chlorophyll binding genes were highly enriched followed by pigment binding, protein binding, and metal ion binding. The analysis using host-specific expression data compared between samples inoculated with *Rpg1* virulent vs avirulent *Pgt* isolates resulted in five genes showing significantly higher expression in samples inoculated with *Rpg1* virulent isolates. Because of this small number of DEGs, we could not perform a gene enrichment analysis for this comparison.

### Differential regulation of fungal genes in *Pgt* with varying virulence profiles

In order to obtain a list of candidates that possibly function as avirulence genes (genes with significantly higher expression in avirulent isolates) or suppressors of resistance (genes with significantly higher expression in virulent isolates), comparative assays were done between groups of samples inoculated with *Pgt* isolates showing differential virulence. The comparative analysis utilizing RPKM expression values from reads mapped to the *Pgt* and barley reference genome sequences yielded DEGs in both comparisons representing RMRL virulent -vs- avirulent and *Rpg1* virulent -vs- avirulent isolates. In the comparisons using reads that mapped to the PASA updated *Pgt* gene models, we found 246 DEGs when using the comparison between virulent -vs- avirulent *Rpg1* isolates (Additional file 1: Table S10). The comparison between RMRL virulent -vs- avirulent isolates identified 428 DEGs (356 exhibited a significantly higher gene expression in the RMRL virulent isolates) (Additional file 1: Table S11). Among the 246 DEGs identified between *Rpg1* virulent -vs- avirulent isolates, 67 genes (55 with relatively higher gene expression in *Rpg1* virulent isolates) were predicted to contain an N-terminal signal peptide and 25 genes (18 with relatively higher gene expression in *Rpg1* virulent isolates) were predicted as CSEPs (Additional file 1: Table S10). Likewise, among the identified DEGs between RMRL virulent -vs- avirulent isolates, 95 DEGs (14 with relatively higher gene expression in RMRL virulent isolates) contained a predicted N-terminal signal peptide and 44 DEGs (6 with relatively higher gene expression in RMRL virulent isolates) were predicted as CSEPs (Additional file 1: Table S10).

Despite showing nearly 400 fungal DEGs, no significantly enriched GO terms were found from the gene enrichment analysis. This problem generally arises when dealing with biotrophic fungal pathogens that are known to have many effector genes that are unique to their own species with very few showing homology to previously characterized genes [43–45]. For example, out of all the annotated gene models in the *Pgt* race SCCL genome sequence, only 37% of the genes are assigned a GO term. These 37% of the *Pgt* genes with known GO terms only represented 24 and 15% of the DEGs observed in the comparison between *Rpg1* virulent and avirulent isolates and RMRL virulent and avirulent isolates, respectively. Due to this small number of gene models with GO terms in the DEG sets, gene enrichment analysis for *Pgt* DEGs was not successful.

### Expression profiling of *Pgt* genes

The overall expression of *Pgt* genes in the isolates used in this RNAseq study was assessed to support the use of a single time point in this study. Of the 15,800 annotated *Pgt* genes, 1710 genes had no detectable expression at 5DPI and were removed from the analysis. Among the remaining 14,121 genes, 54.1% were expressed at extremely low levels, 30.9% were expressed at low levels, 9.3% were expressed at moderate levels, 7.57% were expressed at high levels and 1.3% were expressed at extremely high levels. Among the 2091 *Pgt* genes containing a predicted N-terminal signal peptide, only 20 (0.5%) showed no expression at the time point used for analysis. Among the remaining genes with a predicted N-terminal signal peptide, 66.1, 18.6, 5.4, 7.3, and 2.01% were expressed at extremely low, low, moderate, high and extremely high levels, respectively (Additional file 1: Table S12).

Out of the 2029 *Pgt* genes with a predicted N-terminal signal peptide, EffectorP V2.0 predicted 787 as CSEPs. Only 11 genes encoding CSEPs (1.4%) showed no expression at the 5DPI time point examined. Of the remaining predicted CSEPs, 68.5, 16.5, 4.5, 7.2 and 2.1% were expressed at extremely low, low, moderate, high and extremely high levels, respectively (Additional file 1: Table S12). The observation of expressed genes encoding CSEPs in samples at 5 DPI possibly validates the use of this single time point for capturing putative virulence/avirulence effectors.

### Transcriptome-wide association study

This analysis was done to identify variants in *Pgt* genes that are possibly associated with avirulence or virulence in *Pgt*. A total of 600 K variants (biallelic and multiallelic) in the *Pgt* gene models were obtained after combining all the observed variation from the 24 RNAseq samples. The Ensemble Variant Effect Prediction tool [46] predicted 215 K variants as non-synonymous mutations. All the multiallelic variants were removed and the remaining biallelic variants missing more than 50% of their calls and having a minor allele frequency of less than 1% were filtered out leaving 104 K biallelic variants to be utilized in association analysis. These 104 K variants were distributed across 9122 *Pgt* gene models, with an average of about 11 (S.D. ± 11) variants per gene. Thus, genetic variation resulting in predicted non-synonymous protein changes were identified in 57.7% of the annotated *Pgt* genes. The association analyses were performed using the naïve, Q model, and QK model. The Q model using 3 PCA that explained about 25% of the variation gave comparatively better associations. Upon manual inspection of the variations that resulted in significant marker-trait associations (MTA), it was determined that these MTA appeared to represent false associations. A MAT was considered false when the majority of the *Pgt* isolates within a given virulence group does not share a common allele/SNP call. A manual screening off alleles associated with RMRL virulence/ avirulence was performed that identified 33 variants distributed across 28 gene models that were associated with RMRL specific resistance. Among these 28 gene models, seven of the genes were predicted to contain an N-terminal signal peptide (Additional file 1: Table S13).

## Discussion

The original goal of this research was to identify *Puccinia graminis* f. sp. *tritici* genes representing dominant avirulence factors that interact and elicit defense responses through the *Rpg5* gene, the major R-gene underlying RMRL [31, 32]. These original assumptions followed the central dogma of Flor’s gene-for-gene hypothesis [47], which has held up in characterized Flax-flax rust [37] and wheat-wheat stem rust [36, 38] pathosystem interactions. However, gene expression comparisons of both the host and pathogen during the infection process with a set of RMRL and *Rpg1* virulent -vs- avirulent isolates (Table 2; Additional file 1: Table S13) led to the development of the different hypothesis; dominant virulence genes suppress defense mechanisms in barley that are elicited by the barley stem rust resistance gene *Rpg5* at the RMRL. Here we present two possible scenarios that could determine the outcomes incompatibility (resistance) vs compatibility (susceptibility) in the barley-*Pgt* interactions. First, the lack of avirulence genes represented by 38 putative effector genes with significantly lower gene expression in RMRL-virulent isolates (Additional file 1: Table S9), and/or second; the presence of host defense response suppressor genes in the RMRL-virulent isolates (Tables 1 and 2; Additional file 1: Tables S10 and Additional file 1: Table S13).

**Table 2:** Variants associated with *rpg4/5* specific virulence of *Pgt* on barley*

Gene	SP^a^	Virulent *rpg4/5* isolates^b^									Avirulent *rpg4/5* isolates														
*PGT*G_05610	Y	1	1	1	0	0	1	1	1	1	0	0	0	0	0	0	0	0	0	0	1	0	0	1	0
*PGT*G_07009	Y	1	1	1	1	0	1	1	1	1	0	0	0	0	0	0	0	0	0	0	1	0	0	2	0
*PGT*G_07009	Y	1	1	1	1	0	1	1	1	1	0	0	0	0	0	0	0	0	0	0	1	0	0	2	0
*PGT*G_07009	Y	1	1	1	1	0	1	1	1	1	0	0	0	0	0	0	0	0	0	0	1	0	0	2	0
*PGT*G_08059	Y	1	–	1	–	0	1	–	1	1	0	0	0	0	–	–	0	0	0	0	0	–	0	0	0
*PGT*G_00156	Y	1	2	2	1	0	1	–	2	–	0	0	0	0	0	1	0	–	0	0	0	0	0	0	0
*PGT*G_12898	Y	1	1	1	1	0	1	1	1	1	0	0	–	–	–	1	0	0	0	0	0	0	0	0	0
*PGT*G_13504	Y	1	1	1	0	0	1	1	1	1	0	0	–	0	0	0	0	1	0	0	0	0	0	0	0
*PGT*G_16718	Y	1	1	1	1	0	1	1	1	1	0	0	0	0	0	1	0	0	0	0	0	0	0	0	0
*PGT*G_16718	Y	1	1	1	1	0	1	1	1	1	0	0	0	0	0	1	0	0	0	0	1	1	0	0	0
*PGT*G_10874	Y	1	1	1	1	1	1	0	1	1	0	0	0	0	0	0	0	0	0	0	0	0	0	0	0
*PGT*G_00438	N	1	1	1	1	2	1	1	1	1	2	2	2	2	2	1	2	2	2	2	2	2	2	2	2
*PGT*G_00486	N	1	1	1	1	0	1	1	1	1	0	0	–	0	0	1	0	0	0	1	0	0	–	–	2
*PGT*G_00763	N	1	1	1	1	1	1	0	1	1	0	0	0	0	0	1	0	0	0	0	0	1	0	0	0
*PGT*G_01415	N	1	1	1	1	0	1	0	1	1	0	0	0	0	0	0	0	0	0	0	0	0	0	0	0
*PGT*G_06872	N	1	1	1	1	0	1	1	1	1	0	0	–	0	0	1	0	0	0	0	0	0	0	0	0
*PGT*G_06872	N	1	1	1	1	0	1	–	1	1	0	0	0	0	0	1	0	0	0	0	0	0	0	0	0
*PGT*G_06872	N	1	1	1	1	0	1	–	1	1	0	0	0	0	0	1	0	0	0	0	0	0	0	0	0
*PGT*G_06894	N	1	1	1	1	0	1	0	1	1	0	0	0	0	0	0	0	0	0	0	0	0	0	0	0
*PGT*G_06947	N	1	1	1	1	0	1	1	1	1	0	0	–	0	0	0	0	0	1	0	–	0	–	–	2
*PGT*G_07029	N	1	1	1	1	0	1	1	1	1	0	0	–	0	0	0	0	0	0	0	–	–	–	–	2
*PGT*G_07313	N	1	1	1	1	0	1	0	1	1	0	0	0	0	0	0	0	0	0	0	0	0	0	0	0
*PGT*G_07403	N	1	1	1	1	0	1	1	1	1	0	0	–	0	0	0	0	0	0	0	0	0	–	–	–
*PGT*G_08749	N	1	1	1	1	0	1	1	1	1	0	0	0	0	0	0	0	0	0	0	0	0	0	0	0
*PGT*G_10716	N	1	1	1	1	1	1	1	1	1	0	0	0	0	0	0	0	0	0	0	0	0	0	0	2
*PGT*G_10718	N	1	1	1	1	1	1	1	1	1	0	0	0	0	0	0	0	0	0	0	0	0	2	0	2
*PGT*G_11449	N	1	1	1	1	0	1	0	1	1	0	0	0	0	0	0	0	0	0	0	0	0	0	0	0
*PGT*G_13122	N	1	1	1	1	0	1	1	1	1	0	0	0	0	0	0	0	0	0	0	0	0	0	0	0
*PGT*G_13608	N	2	1	1	2	0	2	1	1	1	2	2	2	2	2	2	2	0	2	2	2	2	2	2	2
*PGT*G_14032	N	1	1	1	1	0	1	1	1	1	0	0	0	0	0	0	0	0	0	0	0	0	0	0	0
*PGT*G_14065	N	1	1	1	1	0	1	1	1	1	0	0	0	0	0	0	0	0	0	0	0	0	0	0	0
*PGT*G_18718	N	1	1	1	1	0	1	1	1	1	0	0	0	0	0	0	0	0	0	0	0	0	0	0	0
*PGT*G_19496	N	1	1	1	1	1	1	1	1	1	0	0	0	0	0	1	0	0	0	0	0	0	0	0	0

*This table is a snippet of Table S13. Refer to Table S13 for detailed information

a- Gene with (Y) and without (N) predicted N-terminal Signal Peptide

0-Homozygous reference, 1-Heterozygoys, 2-Homozygous alternate allele

For the expression and association analysis utilized in this study, a group of thirty-seven *Pgt* isolates collected in the Upper Midwestern United States from 1970 thru the 1990s that exhibited differential virulence on barley lines containing the effective and broad-spectrum resistances conferred by RMRL and *Rpg1* were identified. This was the second study conducted to systematically evaluate seedling reactions of barley lines with different stem rust resistance genes [48]. However, this study utilized mostly uncharacterized rust isolates and surprisingly determined that 22 and 25% of the isolates tested were virulent on *Rpg1* or *rpg4/*RMRL, respectively. This high proportion of isolates containing virulence on these two major resistances was surprising as only single races were previously identified in the US virulent on *Rpg1* [24, 49] or RMRL [48]. Thus, it was previously posited that only a few isolates would contain virulence on these two major genes in barley. We identified several isolates with virulence to either *Rpg1* or RMRL that contained a high level of genome diversity as determined using SNP markers. However, we did not identify any isolates that were virulent on both *Rpg1* and RMRL possibly due to the stabilizing effect on the Midwestern *Pgt* population as a result of the barberry eradication program effectively removing the *Pgt* sexual cycle from the Midwestern US [12].

The RAD-GBS and *in planta* RNAseq analyses allowed for the identification of several *Pgt* isolates with avirulence and/or virulence on barley lines containing either *Rpg1* or RMRL with a high level of genetic diversity. The phenotyping and genotyping data on these isolates were used for comparative and association analyses. The comparative and association analyses in this study were designed based on the hypothesis that virulence/avirulence to a specific R-gene should be governed by a limited number of common effectors that act as avirulence genes or suppressors that are common to isolates that show the same infection types on barley lines carrying the R-gene. Thus, in the comparison between RMRL virulent-vs-avirulent isolates, the experimental replication was built into the number of isolates sharing the common virulence profiles (*N* = 9 RMRL virulent and *N* = 15 RMRL avirulent isolates) instead of running multiple replications of RNAseq on the same isolate. The use of multiple diverse isolates classified based on their interaction with the specific host R-gene provides significance to the analysis to avoid making a type I error. However, such an experimental design may lose power when the population contains different effectors that can lead to a common virulence/avirulence phenotype. For example, if a pathogen population contains two different effectors that can suppress the same resistance mechanism.

A single time point (5DPI), that had shown effector expression during the colonization process [17] was used for analysis. A previous time-course transcriptomics study on stripe rust inoculated wheat showed an increasing trend of fungal reads starting at 5DPI, suggesting that 5DPI was a suitable time point for this study [17]. Based on extensive phenotypic observations across the stem rust pathogen’s colonization process in barley, the first macroscopic sign of successful *Pgt* infection are visible at ~ 4-5DPI which allowed for the collection of leaf samples displaying multiple infection sites. The fact that 98.6% of the predicted *Pgt* candidate secreted effector proteins (CSEPs) were expressed in at least some isolates analyzed (Additional file 1: Table S12) at 5DPI demonstrated that a time point was captured with the expression of most of the secreted effectors.

The compatible (susceptible) host variety Harrington was utilized so the RNAseq libraries would provide a somewhat balanced proportions of mRNAs from both the host and pathogen after infection with each of the *Pgt* isolates utilized. As expected, a balanced number of reads representing both the fungal pathogen as well as the host were generated, facilitating transcript analyses of both at this time point. Thus, the transcriptomics data generated at this single time point on the susceptible variety Harrington would be sufficient to achieve the objectives of identifying *Pgt* effectors that function as avirulence or virulence genes when interacting with the *Rpg1* or RMRL by analyzing differential gene expression and genetic diversity in virulent and avirulent interactions from both the pathogen and host perspectives.

Despite having a group of isolates with balanced avirulence-virulence profile for both -RMRL (9 out of 24 isolates) and *Rpg1* (8 out of 24 isolates), and samples collected at a time point that contained a high proportion of fungal transcripts, the AM analyses utilizing the expression data did not produce informative marker-trait associations between the phenotypic and genotypic variation. The AM using the disease reaction of the 24 *Pgt* isolates on HQ1 (RMRL*; rpg1-*) produced MTA with low significance spread throughout the *Pgt* genome representative of background noise with no highly significant marker-trait associations for RMRL virulence/avirulence. Similarly, the AM analysis using infection types on cv Morex (rmrl-; *Rpg1+*), showed no MTA with *Rpg1* virulence/avirulence. A study similar to the present research was conducted to identify avirulence effectors of *P. striiformis* f. sp. *tritici* (*Pst*) using 14 diverse *Pst* isolates with different virulence/avirulence profiles for 18 yellow rust resistance (*Yr*) genes [19]. That study identified candidate *Avr* effector specific to six *Yr* genes but failed to obtain significant MTA associated with the five other *Yr* genes (*Yr7*, *Yr27*, *Yr43*, *Yr44*, and *YrExp2*), despite having balanced virulence-avirulence profiles, similar to the results observed in our study. In the present study, the absence of MTA can probably be attributed to the small population size and the *Pgt* populations in the Great Plains of United States are mostly clonal asexual populations that resulted due to the eradication of the alternate host barberry [12, 49, 50]. The clonality in the population can have a big effect on the accuracy of determining linkage disequilibrium as the individuals within the clonal population retain a high level of heterozygosity that can reduce the power of AM, leading to the difficulty of detecting significant MTA and the identification of multiple MTA with low significance representing false positive noise where it is difficult to sift out the true MTA [19, 51]. A transcriptome-wide association study utilizing a smaller, but geographically diverse set of 17 powdery mildew fungal isolates identified two candidate effectors, *Avr*_*a1*_ and *Avr*_*a13*_ that are recognized by the cognate powdery mildew resistance gene alleles *Mla1* and *Mla13*, respectively. Suggesting that fungal isolates of diverse origin give better results in association mapping studies [23]. However, noisy associations like that observed in this AM are not uncommon, and can also be attributed to the presence of multiple effectors that have specificity for a given *R*-gene [19]. The differential effector interactions with the same host resistance mechanisms adds complexity to the association leading to reduced significance of the positive MTA. The greater possibility of identifying more false positives is due to the reduced significance of the true MTA leading to more false negative results as the positives become lost in the background noise of the false positive MTA. Thus, if isolates contain different effectors that interact with a single *R*-gene, then MTA for each single effector may be insignificant as the significance of the single gene interaction would be lost due to the variant allele and associated MTA not being shared or supported by other avirulent isolates, especially when a small population was utilized for the analyses. A shortcoming of the AM analysis software in studying transcriptome based mapping fungal pathogen is the lack of variants from non-coding region and absence of sexual recombination that can lead to a false or improper estimation of linkage disequilibrium leading to false-positives [19].

Due to the lack of apparent association between virulence and genotype, the genotypic data were manually inspected to find some false negative variants associated with virulence/ avirulence for *rpg4*. The reference genome used in this study was generated from *Pgt* race SCCL [39], which is avirulent on *Rpg1* or RMRL containing barley lines ([52]; personal communication: Brian Steffenson, University of Minnesota, MN). This would suggest that the reference genome contains avirulent alleles specific to *Rpg1* and RMRL. So, virulent isolates should carry the alternate alleles (alleles different from SCCL) for genes encoding *Avr* effectors and/or suppressor of resistance. An important point concerning the results of our analysis is that if a dominant suppressor of resistance governs the virulence, then the virulent isolate should have the suppressor allele in either a homozygous or heterozygous state.

A manual screening of each variant was done to identify variant/s carrying an alternate allele, either in the heterozygous or homozygous state for more than 75% of virulent isolates (maximum of 2 outlier calls) and homozygous reference allele for more than 80% of the avirulent isolates (maximum of 2 outlier calls). No maker trait associations were evident with the *Rpg1* specific virulence data; however, thirty-three variants were identified that were associated, 78 to 100% with virulence on RMRL*.* These 33 variants were within 28 different gene models, among which seven were predicted to contain a predicted N-terminal signal peptide (Additional file 1: Table S13). Interestingly, most of the variants had a heterozygous genotype for the RMRL virulent isolates and were homozygous for the alternate allele in the avirulent isolates (Additional file 1: Table S13). The heterozygosity in a pathogen, especially at avirulence loci was suggested to result from a positive selection that favors adaptive fitness [19] and plays a role in progressive virulence [53]. The presence of heterozygosity at the putative virulence loci in isolates with specific virulence on RMRL suggests that the genes that encode the avirulence protein that interacts with Rpg5 (The R-protein at RMRL) are possibly present in the majority of *Pgt* isolates and have an essential virulence function that cannot be disposed of, possibly representing a PAMP. Thus, the pathogen was pressured to evolve dominant virulence gene(s) that suppress the RMRL resistance response, which manifests itself genetically as a dominant virulence gene.

Effector proteins that function to suppress *R*-gene-mediated resistance upon recognition of the cognate avirulence protein have been identified for the biotrophic fungal pathogen *Blumeria graminis* the causal pathogen of powdery mildew [54, 55]. This study with the wheat-powdery mildew pathosystem identified two *B. graminis* genes, *AvrPm3*^*a2/f2*^ an avirulence gene and *SvrPm3*^*a1*^ a suppressor of avirulence, that specifically interact with the wheat powdery mildew resistance gene *Pm3f* [54]. *Pm3f* based resistance was observed only in the isolates containing functional *AvrPm3*^*a2/f2*^ and a non-functional *SvrPm3*^*a1*^*.* In rust pathosystems, it was also shown genetically that some strains of *Melampsora lini,* the causal pathogen of flax rust [56] are known to carry an inhibitor of avirulence/R-gene resistance responses that was designated the *I* gene. The *I* gene inhibits the HR-mediated resistances elicited by avirulence genes, *AvrL1*, *AvrL567*, *AvrL8*, *AvrL10*, and *AvrM* when recognized by the corresponding *R*-gene alleles, *L1*, *L7, L8*, *L10*, and *M1,* respectively [56]. However, the *I* gene has yet to be identified. A recent study of the wheat stripe rust pathosystem conducted to characterize the host-pathogen genetic interactions that determine *P. striiformis* f. sp. *tritici* virulence on wheat*,* the causal agent of stripe rust, identified dominant virulence loci in *Pst* isolate 08–220 that corresponded to the suppression of the yellow rust resistance gene *Yr1, Yr6, Yr7, Yr8, Yr9, Yr21, Yr25, Yr27, Yr28, Yr35, Yr8, Yr35, Yr41, Yr43, Yr44, Yr76, YrA or Yr74* [57]. Interestingly, going into the study to characterize these interactions with dominant *Yr* resistance genes it was expected that they would represent dominant *R*-gene-Avr gene interactions. However, to the author’s surprise, almost all the interactions were explained by dominant virulences or recessive avirulence gene interactions suggesting the presence of dominant suppressors of resistance. All of these findings within biotrophic pathogen-cereal host pathosystems suggest the presence of suppressors/inhibitors of avirulence (*Svr*/*I*). Thus the data from these other pathosystems supports the hypothesis that in our barley-stem rust pathosystem, avirulence may be determined by the absence of a dominant virulence effector or suppressor of immune responses similar to what has been recently reported for the wheat-stripe rust pathosystem.

Among the list of candidate genes with variants associated with virulence (Additional file 1: Table S13), the gene model *PGT*G_06872 was particularly interesting because it is predicted to encode a protein phosphatase 2C (PP2C) protein. Barley lines harboring the *rpg5* allele containing the PP2C integrated sensory domain, *rpg5*-PP2C, are susceptible to *Pgt* races. The functional *Rpg5* alleles contain a serine-threonine protein kinase (STPK) integrated sensory domain and this allele is required to provide broad resistance against the majority of *Pgt* races, including the North American *Rpg1* virulent race QCCJB and the highly virulent African race TTKSK and its lineages [29–31]. However, when the *Rpg5-STPK* and *rpg5-PP2C* alleles are present together in the heterozygous state it was determined that the *Rpg5-PP2C* allele functions as a dominant suppressor of the otherwise dominant *Rpg5-STPK* resistance response suggesting that the suppressor action explains the previously reported recessive nature of *rpg4*-mediated wheat stem rust resistance [58]. Although the putative fungal effector containing the PP2C domain does not have significant homology to the *rpg5* PP2C domain it is an attractive candidate for a suppressor of RMRL resistance. It could be speculated that *PGT*G_06872 PP2C could antagonize the *Rpg5 STPK*-mediated phosphorylation events underlying *rpg4*-mediated resistance. Thus, the variation or mutation in *PGT*G_06872 may have allowed this allele to function as a dominant suppressor of RMRL resistance in virulent isolates. However, *PGT*G_06872 does not contain a signal peptide or transmembrane domain suggesting that it may not represent a secreted effector.

Another objective of this study was to conduct a comparative analysis of barley gene expression in response to virulent and avirulent *Pgt* isolates. For the host-specific comparative analysis, the universal stem rust susceptible barley line Harrington was selected for this study. Harrington is susceptible to all the isolates used in this study because it does not carry either *Rpg1* or RMRL. Although, inoculating the resistance line with virulent and avirulent isolates for comparison of host transcripts during compatible and incompatible interactions may have been informative in regard to host defense responses, the major objective of this study was to identify fungal effectors/elicitors by studying differentially expressed fungal transcripts when comparing diverse virulent and avirulent isolates. To this end, the use of a resistant line would have affected the recovery of fungal transcripts from the avirulent isolates. Thus, to fulfill our major objective it was more informative to inoculate the isolates on a susceptible genotype independent of the strong resistance responses elicited by the resistance genes as it allowed for the determination of differential pathogen genes elicited between the *Rpg1* and RMRL virulent and avirulent isolates. Thus, to accommodate our major objective this study was conducted using the susceptible barley line Harrington.

A successful pathogen is able to not only surpass early PTI responses but also evolve effectors that it releases into the host cells to manipulate host physiology to facilitate avoidance of ETI responses and acquisition of nutrient to support colony growth and ultimately reproduction [1]. Avirulent pathogens carry the effector/s that is specifically identified by a host R-protein, while virulent pathogens lack the avirulence effector and/or carry suppressors of resistance to negate the *R*-gene based recognition [10, 56]. In this analysis, the use of the susceptible line Harrington does not significantly impede the infection process of any of the *Pgt* isolates used, thus, all isolates should be able to release their effectors/elicitor repertoire to manipulate the host which should include avirulence proteins as well as proteins that evolved to suppress R-gene mediated resistance responses, even in the absence of the cognate R-gene. The differential expression of host genes during their interaction with diverse sets of virulent and avirulent isolates in respect to *Rpg1* or RMRL in the absence of the corresponding resistance genes implies that the differential host gene expression is in response to specific polymorphic effector/s between virulent and avirulent isolates. Thus, we hypothesize that the differences in the effector profile in the sets of diverse RMRL virulent and avirulent isolates could induce or suppress basal defense-related genes in the susceptible line Harrington even in the absence of the specific R-gene.

Interestingly, the comparative analysis of host specific transcripts between samples inoculated with RMRL virulent and avirulent isolates showed a set of 115 high confidence barley DEGs specific to the interactions with RMRL virulent *Pgt* isolates, with an even split of 58 genes that exhibited a significantly higher expression and 57 genes with significantly lower gene expression in samples inoculated with RMRL virulent isolates (Additional file 1: Table S10). In contrast, when the analysis was performed by separating the groups into *Rpg1* virulent -vs- avirulent isolates, only five high confidence DEGs were detected and they had a significantly higher expression in *Rpg1* virulent inoculated samples. Due, to this significant number of DEGs being induced specifically by the RMRL virulent isolates and very few DEGs observed in the comparison between *Rpg1* virulent and avirulent isolates, as well as no association being identified for *Rpg1* virulence, the remaining discussion will focus on RMRL virulence and avirulence. To validate that the 57 host genes that exhibited a suppressed gene expression during the interaction with RMRL virulent isolates, a comparative assay of barley gene expression was performed between RMRL virulent *Pgt* inoculated-vs-non- inoculated controls and RMRL avirulent *Pgt* inoculated-vs-non- inoculated controls. The analysis confirmed that these under expressed genes were in fact expressed at higher levels in the RMRL avirulent inoculated samples compared to the non-inoculated samples; while the expression levels were not significantly different between the RMRL virulent inoculated and non-inoculated control samples. Thus, these expression analyses support the hypothesis of a virulence factor or factors in RMRL virulent isolates that potentially suppress and induce the expression of multiple genes during their host-pathogen interaction (Additional file 1: Table S10).

The host genes suppressed in response to the RMRL virulent isolates were enriched for heat shock proteins (HSPs), with the majority in the class of small HSPs (sHSPs) and HSP70. It had been shown previously that sHSPs are involved in HR-independent non-host type basal immune response by studying the pathogenic *Ralstonia solanacearum* OE1–1 and the non-pathogenic *R. solanacearum* 8107 strains when infecting tobacco [59]. Similar studies were also performed with the bacterial pathogens *Xanthomonas axonopodis* pv. *citri* and *Xanthomonas campestris* pv. *vesicatoria* that cause citrus canker and bacterial spot of pepper, respectively [60]. In barley, two powdery mildew candidate genes CSEP105 and CSEP0162 were shown to contribute in virulence by interfering with the chaperone activity of barley small heat shock protein, Hsp16.9 and Hsp17.5 [42]. We also observed virulent isolate specific suppression of Hsp16.9, Hsp17.6, and Hsp17.9 (Table 1). These studies showed that the induced expression of the sHSP was a PAMP triggered immunity (PTI) response [60]. Strikingly, HSP70 that has been expressed at significantly lower level in response to virulent RMRL isolates in this study has been shown in many studies as a key player in HR-mediated ETI resistance responses and are targeted by several pathogenic effectors to gain virulence [41, 61–64]. The bacterial pathogen *Pseudomonas syringae* is also able to induce virulence by utilizing a virulence effector HopI1 to target an *Arabidopsis* HSP70 protein [41]. The oomycetes pathogen *Phytophthora sojae* utilizes the effector PsCRN 108 (Crinkler or crinkling- and necrosis-inducing protein) to target a HSP promoter to suppress its expression [64].

In mammalian pathosystems, there is a parallel function of suppression of HSP proteins to induce virulence. The transcriptional regulation of *Hsp70* is dependent upon the binding of the heat shock factor (HSF) to a specific DNA component in heat shock element (HSE) [65]. The expression of a heat shock factor − 3 (HSF3) was found to be regulated by direct binding to the c-myb proto-oncogene product (c-MYB) suggesting a role of c-MYB in transcriptional activation of *Hsp70* [66]. Another study has shown that a tumor suppressor protein p53 can modulate the expression of *Hsp70* by binding to HSF3 and disrupting the c-MYB/HSF3 association [66], suggesting that suppressors can act on either component of the c-MYB/HSF3 complex to inhibit HSP70 expression. These findings show that HSPs can be suppressed at different levels of transcriptional regulation possibly presenting different targets of effector manipulated to suppress HSP proteins and immunity responses. In plants, the R2R3 MYB-proteins are the classical MYB factors that are close homologs to c-MYb in mammals [67]. Interestingly, the RMRL virulent *Pgt* isolates significantly suppressed a host gene encoding a myb domain that could partially explain the observed lower expression of the *Hsp* family of genes in the barley line Harrington (Additional file 1: Table S10).

Another interesting aspect of the host DEGs was the predicted subcellular locations where their protein products function. Gene enrichment analysis for cellular function showed that the majority of both higher and lower expressed genes were chloroplast-localized proteins (Additional file 1: Table S11). The chloroplasts play a major role in plant defense response by producing several reactive oxygen species (ROS), which are involved in both signal transduction and HR-mediated resistance [68]. Thus, evolutionarily it may be beneficial for pathogens to evolve effectors that can subvert chloroplast function and suppress chloroplast ROS production. The relatively lower expression of genes encoding ferredoxin, ribulose bisphosphate carboxylase (rubisco), NAD(P) H dehydrogenase subunit H, and Photosystem II protein D1 (Additional file 1: Table S10) [69, 70] suggests that virulent RMRL isolates may have evolved suppressors that target defense responses that once activated provide induction of chloroplast function.

During the molecular arms race between rust pathogens and their host, the main hypothesis concerning the pathogen’s ability to gain virulence once detected by a cognate specific *R-* gene is to evolve avirulence gene diversity such that recognition is lost following Flor’s classic gene-for-gene model [47]. However, it is also possible that the *Avr* gene recognition could be lost via gene deletion through unequal recombination or mutations that result in loss of expression. To determine if *Pgt* virulence was possibly due to the loss of an avirulence gene or transcriptional silencing, transcriptome analysis was done to identify differentially expressed CSEPs. This study identified 38 CSEPs that were expressed at much lower levels in RMRL-virulent isolates, and thus the possibility cannot be ruled out that the virulence of these isolates is due to loss or differential regulation of an effector/avirulence gene(s) and these downregulated genes represent candidate RMRL avirulence genes (Additional file 1: Table S9). However, six effector genes expressed at higher levels in the RMRL virulent isolates identified from the same analysis are also candidate suppressors of RMRL (Additional file 1: Table S9).

**Fig. 4:**
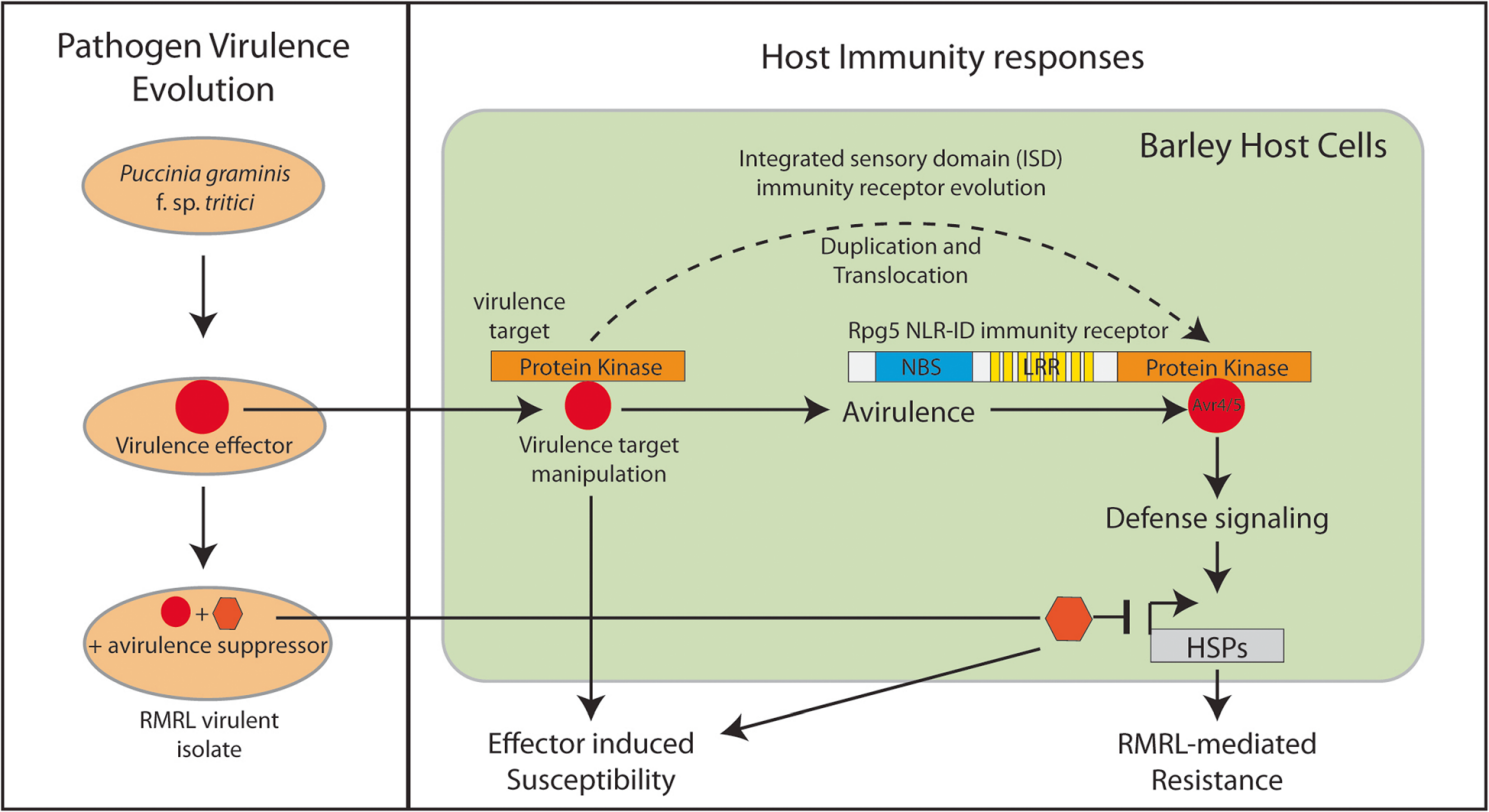
A model showing the hypothetical evolution and mechanisms of *Puccinia graminis* f. sp. *tritici* (*Pgt*) effector interactions and function to elicit and suppress the barley RMRL-mediated resistance mechanism. The box on the right shows the stepwise evolution of virulence effectors that allows *Pgt* to become virulent on barley (virulence effector depicted by a red circle) then subsequently repress RMRL mediated resistance through gaining a suppressor of Avr4/5 avirulence. The host immunity evolution model shown in the box on the left was developed based on the genetic and functional analyses of RMRL that shows it represents an integrated sensory domain (ISD) nucleotide binding site-Leucine rich repeat (NLR) resistance locus [58]. The barley NLR *Rpg5* contains a Protein Kinase domain that represents an integrated sensory domain (ISD) that recognizes the Avr4/5 avirulence effector that originally evolved as a virulence effector that targets the progenitor Rpg5 protein kinase as a virulence target. Based on the ISD hypothesis we speculate that the *Pgt* virulence effector targeted the protein kinase in barley to gain effector induced susceptibility (EIS). As these susceptibility targets represent host proteins with a critical function barley was forced to co-evolve by duplicating and translocating this susceptibility hub to the *Rpg5* NLR that functions as pathogen “bait” that recognizes the virulence effectors manipulation of the original susceptibility target through its ISD triggering RMRL-mediated resistance. Thus, the original virulence effector is now transformed by host evolution to the avirulence effector Avr4/5. During the molecular arms race, the pathogen may be unable to shed Avr4/5 as it has an essential virulence function, thus evolved a suppressor of RMRL-mediated resistance signaling which includes the heat shock proteins and others (Table 1; Additional file 1: Table S10) to regain EIS

Understanding and developing hypotheses explaining the complex host-pathogen genetic interactions occurring in this pathosystem requires information from both the host and pathogen perspective. Thus, information recently discovered on the putative function of the genes underlying the RMRL-mediated resistance mechanisms suggest the “integrated sensory domain (ISD) hypothesis” is responsible for the evolution of this resistance mechanism [58]. Genome analysis of several plant species [71, 72] suggests that the two NLR genes present at the RMRL, *Rpg5* and *HvRga1*, fit the role of dual plant NLR immunity receptors found in the tightly linked head-to-head genome architecture with one NLR containing a non-canonical domain that represents a pathogen virulence effector target. Recent, functional analyses of the RGA4/RGA5 proteins that confer resistance to *Magnaportha oryzae* determined that one NLR contains an ISD that represent a virulence effector target that was translocated to the immunity receptor [73], possibly via a targeted mechanism mediated by the dual NLR architecture [74]. The barley NLRs at the RMRL, *Rpg5* and *HvRga1,* are both required for resistance and are present in the head-to-head genome architecture suggesting that the STPK domain that was putatively translocated to the *Rpg5* NLR immunity receptor possibly had an original function in normal physiological processes that the stem rust pathogen hijacked to facilitate virulence. Thus, the STPK ISD may function as a pathogen “bait” that is targeted by a virulent effector, now effectively an avirulence gene that initiates RMRL-mediated defense responses. We posit that the host (barley) evolved to translocate these susceptibility hubs to NLRs because on the host side they have an essential function and the effector that evolved in the pathogen to manipulate this susceptibility target in the host is essential for host-specific pathogenicity. Thus, in our working model we speculate that both virulent and avirulent isolates may contain the *Avr-*RMRL effector, yet, the virulent isolates evolved an effector that suppresses RMRL resistance mechanisms leading to virulence on RMRL (Fig. 4).

The hypothesis that Avr-RMRL may be a conserved effector present in both virulent and avirulent *Pgt* isolates which targets the function of the progenitor protein of the Rpg5-STPK ISD suggests that the RMRL avirulence effector, Avr-RMRL, may be conserved across *Pgt* isolates thus can’t be detected via functional polymorphisms or expression differences. Thus, the gain of virulence-specific for RMRL may be guided by the presence of virulent effectors/suppressors in the RMRL virulent isolates. The association mapping indeed did identify putative effectors that have gene action supporting the dominant virulence model. This model is also supported by the fact that a significant number of host genes involved in resistance responses are suppressed specifically by the RMRL virulent isolates. Most interestingly, one of the putative effectors that could be involved in suppression is a PP2C. This may be significant due to our recent finding that the barley *rpg5*-*PP2C* allele is able to suppress the otherwise dominant *Rpg5-STPK* allele leading to susceptibility against the majority of *Pgt* isolates [58]. Thus, this putative effector’s predicted gene function suggests that it could utilize a similar mechanism to block RMRL*-*mediated resistance. However, there remains major knowledge gaps in the mechanisms underlying this pathosystem as well as in the understanding of suppressor or inhibitor of avirulence (*Svr* or *I)* and we still need to validate the proposed model. The recent genetic evidences of inhibitor genes in flax rust [56], virulence loci in stripe rust [57] and suppressor of avirulence in powdery mildew [54] suggest that the gain of virulence by acquiring a functional *Svr/I*, instead of losing an *Avr* effector could be more common in pathosystems than previously thought. Similarly, it may be that RMRL virulence is governed by the presence of a suppressor, but our results also showed that we identified candidate avirulence genes suggesting that virulent isolates may also lack the avirulence effectors. The transcript analysis presented in this study filled some gaps in our knowledge that allowed us to develop a model (Fig. 4) to move the hypothesis-driven research forward from both the host and pathogen perspectives to begin elucidating the mechanisms of host-pathogen interactions that result in compatible -vs- incompatible interactions in this complex RMRL stem rust resistance mechanism.

## Conclusions

The understanding of the original virulence function of effectors and the co-evolutionary events that result in avirulence due to *R*-gene specificity is important for the deployment of durable resistance. Sequencing technology coupled with powerful bioinformatics tools has made it possible to begin dissecting these host-pathogen interactions from both the host and pathogen perspective to begin forming hypotheses that will require further functional validation. In this study we were able to utilize several bioinformatics tools with RAD-GBS and RNA sequencing data to begin the process of developing models of *Pgt* virulence/avirulence on an important barley resistance mechanism. We hypothesize that the pathogen has evolved to gain virulence by suppressing effector elicited RMRL resistance responses and the virulence factors are possibly able to impede downstream signaling of resistance responses after early pathogen recognition. Alternatively, the virulence factors could possibly inhibit the interaction between resistance and avirulence genes to suppress the *R*-gene mediated responses. This study significantly added to previous knowledge allowing for the development of a hypothetical model for how *Pgt* gains virulence specific to the important RMRL resistance mechanism in barley, setting the stage to begin functionally validating the candidate genes, from both the pathogen and host that contribute to post-pathogen interactions that result in compatible or incompatible interactions.

## Materials and methods

### Plants, pathogens and inoculation

Six different barley varieties, accessions or recombinant lines, Q21861, HQ1, Harrington, Morex and Steptoe, were used in this study. Q21861 is an unimproved barley line that contains the two wheat stem rust resistance genes, *Rpg1* and *rpg4* [28]. After genetic characterization of the genes required for *rpg4*-mediated resistance, the genes or more appropriately the locus containing the three genes required for wheat stem rust resistance is now referred to as the *rpg4-*mediated resistance locus, RMRL [31]. HQ1 is a near-isogenic line with the Q21861 RMRL introgressed into the susceptible cultivar Harrington background [29] that was developed through backcrossing and marker-assisted selection. Chevron (PI 38061) is a cultivar derived from an unimproved bulked seed lot imported by U.S. Department of Agriculture Switzerland in 1914 [75]. It is the main source of *Rpg1* in North American barley cultivars. Morex is the source of *Rpg1* from which the gene was identified via a positional cloning approach [75]. Both Harrington and Steptoe are wheat stem rust susceptible lines that do not harbor any known stem rust resistance genes [29]. The barley lines used in this study were kindly provided by Dr. Andris Kleinhofs, Washington State University.

A collection of stem rust samples collected during cereal rust surveys conducted led by Dr. James Miller in North Dakota from 1977 to 1999 was maintained as single pustule isolates in the United States Department of Agriculture - Agricultural Research Service (USDA-ARS), Fargo, ND, USA (Pers. Comm. Dr. Timothy Friesen, USDA-ARS Fargo, ND, USA). Thirty-Seven *Pgt* isolates were randomly selected from this large collection, kindly provided by Dr. Timothy Friesen. All of these isolates were increased in an isolation chamber by inoculations on the susceptible barley variety Steptoe with urediniospores collected at 12 – - 14 days post inoculation (DPI). The inoculations were performed as explained by [30]. The virulence of most of these isolates on barley lines containing the wheat stem rust resistance genes *Rpg1* and RMRL were not known and were characterized in this study using the aforementioned barley accessions.

Each of the three independent replications of the virulence assays contained seven individual barley seedlings of each line (Q21861, HQ1, Harrington, Morex, and Steptoe) grown in separate cone containers. The seedlings were grown in a growth chamber (Model 7301-75-2; Caron, Marietta, OH, USA) set at a 16/8-h light/dark cycle and day/night temperatures of 21/18 °C. Seven days after planting, the seedlings were inoculated using the previously established protocol described by [30]. Immediately following inoculation, the seedlings were placed in the humidity chambers with intense light provided by a high-pressure sodium lamp for 18 h. Then the seedlings were returned to the growth chamber set at the previously described condition for 12–14 days. The infection types (ITs) were assessed 12–14 days post inoculation (DPI) using a modified 0–4 scale as previously described [30, 76]. The scale was slightly modified from that developed by [76] as barley exhibits slightly different responses including more mesothetic reaction types [30, 77].

### DNA extraction and RAD-GBS

Restriction Site Associated DNA-Genotyping by Sequencing (RAD-GBS) was done for the 37 *Pgt* isolates to identify variants (SNP/INDEL) amongst the isolates and the *Pgt* race SCCL reference genome [39, 40] and assess the genetic diversity between these isolates. The fungal genomic DNA for RAD-GBS library construction was extracted directly from rust spores using the PowerPlant® Pro DNA (MO BIO Laboratories, Inc.) extraction kit with slight modification to the manufacturer’s protocol to meet the fungal genomic DNA extraction needs. Approximately 30 mg of fungal spores were transferred to 2 ml PowerPlant® Bead Tubes provided with the kit and pre-crushed in a Mixer Miller Type 301 tissue grinder (Retsch Gmbh & Co. KG, Germany) set at a frequency of 30/s for 2 min. The samples were mixed with 410 μl of PD1 solution and 60 μl of Phenolic Separation Solution to disassociated phenolics from the DNA that was later removed by the Inhibitor Removal Technology® step. About 3 μl of RNase was added to the mix and heated for 15 min at 65 °C. The heated samples were again crushed in Mixer Miller Type 301 tissue grinder set at a frequency of 30/s for 4 min and centrifuged at 13,000 x g for 2 min. The supernatant was transferred to clean 2 ml collection tubes provided in the kit and 210 μl of PD3 solution was added and vortexed. The mix was incubated at 4 °C for 5 min and the remaining steps were followed as recommended in the kit’s manual. DNA concentrations were measured using the Qubit® High Sensitivity DNA kit with the Qubit® 2.0 fluorometer (Invitrogen, Carlsbad, CA, USA). A total of 200 ng of DNA from each isolate was used to construct a RAD-GBS library as described by [78]. The 37-isolate specific barcoded libraries were size selected for 200 bp, 240 bp, 275 bp and 300 bp by loading 30 μl; per lane from the 120 μl total library using separate 2% agarose cassettes with the Pippin Prep (Sage Science, Beverly, MA, USA) size selection system set with the narrow selection option for each fraction collected. Four different sized libraries were loaded separately on Ion Torrent 318™ Chips v2 and sequenced using the Ion Torrent Personal Genome Machine® (PGM™) System.

### Diversity assaying using RAD-GBS data

The sequences obtained from RAD-GBS were parsed by barcodes to obtain sample-specific sequences for polymorphism analysis between the isolates. For each *Pgt* isolate, four different FASTQ files sequestered by barcodes representing each different size fraction library were combined for the analysis. The sequence reads were trimmed for quality using default settings in CLC Genomics Workbench 8 (QIAGEN). The quality reads from each isolate were aligned to the *Pgt* race SCCL reference genome sequence v2.0 [39, 40] using the Burrows–Wheeler Aligner maximal exact match (BWA-MEM) algorithm [79]. The variant calling was done using GATK UnifiedGenotyper with the default setting for multi-sample Single Nucleotide Polymorphism (SNP) calling [80]. VCFtools was used to remove individual calls with a read depth of less than six and genotype quality less than ten [81]. Variants, SNPs and Insertions/Deletions (INDELs) with LowQual FLAG were removed from the dataset. A minor allele frequency cutoff of > 1% and missing data cutoff of < 50% was used to select variants for the diversity assay.

The high quality and polymorphic variants identified from RAD-GBS was used to assess the diversity within the 37 isolates used in this study. The familial relatedness (kinship matrix) between the isolates was assessed by computing identity by state (IBS) and hierarchical clustering of the isolates with the fast ward method JMP® Genomics v8.0.

### RNAseq library preparation and sequencing

The RNAseq study was conducted to identify differentially expressed pathogen and host genes by comparing groups of *Pgt* isolates with different virulence profiles, as well as to identify variants in pathogen genes for association analysis with the virulence phenotypes. To facilitate the comparison of expressed *Pgt* genes corresponding to the virulence profile, the universal susceptible barley variety [23] Harrington was used that would allow all *Pgt* isolates, regardless of their virulence profile on *Rpg1* and/or RMRL, to infect and colonize the sampled leaves. The use of the susceptible line Harrington without the R-genes was justified because in most pathosystems analyzed virulence effectors evolve in the pathogen to target host virulence target genes/proteins to become adapted pathogens. These effectors are typically expressed during the infection process regardless of whether the cognate resistance gene is present in the line or not. The plant counteracts these interactions by evolving resistances to recognize these interactions that cause virulence thus the effectors become avirulence genes in the presence of the cognate resistance. Thus, in experiments where one is mainly focused on characterizing the effector genes expressed during the infection process it would not be prudent to use a line containing the R-gene/s as it stops pathogen growth inhibiting the ability to characterize their effector repertoires.

Twenty-four comparatively diverse *Pgt* isolates were utilized to construct RNAseq libraries for the identification of variants (SNP/INDELs) within these isolates as well as to analyze host and pathogen differential gene expression for comparative analysis. *Pgt* isolates were inoculated individually on susceptible barley cv. Harrington for the *in-planta* RNAseq analysis. Nine seven-day old cv Harrington seedlings grown in separate plastic containers were swab inoculated using a cotton bud soaked with Soltrol containing 20 mg of freshly collected urediniospores/ml of each isolate. The inoculated seedlings were incubated in humidity chambers at 100% relative humidity for 18 h following the previously established protocol described in [30]. The inoculated seedlings were moved to isolation chamber in the greenhouse to allow the colonization process to proceed. At five DPI six primary leaves were collected per isolate and immediately frozen in liquid nitrogen and placed in a − 80 °C freezer until RNA isolation was conducted for *in-planta* RNAseq. The remaining seedlings were left in the isolation chambers until fourteen DPI to evaluate infection and efficiency of inoculations. Three replications of uninoculated cv. Harrington were also collected to obtain uninoculated control RNAseq data.

Total RNA was extracted from the inoculated leaves using the RNeasy mini kit (Qiagen, Chatsworth, CA). RNA concentrations were measured using the Qubit® Broad Range RNA kit on a Qubit® 2.0 fluorometer. The quality of the RNA was assessed with an Agilent 2100 Bioanalyzer (Agilent Technologies, Palo Alto, CA, USA). Three inoculated leaves of equal size (~ 2 cm long) per isolate were combined in a single tube and used for total RNA extraction. About 1 μg of total RNA was used for RNAseq library construction using the TruSeq RNA Library Prep Kit v2 (Illumina, San Diego, CA) following the manufactures standard protocol. The final library was validated and quantified on the Agilent 2100 Bioanalyzer. One cDNA library per treatment (No of replication of per isolate = 1) was obtained to process for RNA sequencing. Two library pools, each representing 12 different cDNA libraries were prepared and normalized according to the manufacturer’s protocol. Each of the library pools was diluted to a concentration of 1.8 pm and sequenced on the Illumina NextSeq 500 sequencer on a single flow cell at the USDA Cereal Genotyping Centre, Fargo, ND, USA. To generate 150 bp single-end sequencing reads, the NextSeq® 500/550 High Output Kit v2 (150 cycles) was used. The raw sequencing reads were demultiplexed and converted into individual fastq files using bcl2fastq software v2.17.1.14 (Illumina, San Diego, CA). The fastq reads were quality trimmed in CLC Genomics Workbench v8.0.3 (CLC bio, Aarhus, Denmark) using default settings.

### Program to assemble spliced alignment annotation

The transcriptomic data obtained from this study were used to update the publicly available protein-coding gene annotation of the reference isolate *Pgt* race SCCL [40] using the eukaryotic genome annotation tool PASA (Program to Assemble Spliced Alignment) [82, 83]. A comprehensive transcriptomic database was created using the results from Trinity (v2.4.0) genome-guided RNA-seq and de novo [83, 84]. The trinity genome-guided RNA-seq assembly requires coordinate-sorted bam files. The transcriptomic data from each sample were mapped to the reference genome sequence of *Pgt* race SCCL using the RNA-seq alignment software STAR to generate bam files that were sorted by coordinates (explained below in section ‘Variant calling from RNAseq data’). The bam files from all 24 samples were merged using ‘samtool merge’ to obtain merged coordinate-sorted bam files for Trinity genome-guided assembly [85].

The Trinity de novo assembly was done using the unmapped reads to both the *Pgt* and barley genomes. The high quality trimmed sequencing reads were mapped to the *Pgt* race SCCL reference genome sequence [39, 40] and barley RefSeq v1.0 [86] in CLC Genomics Workbench v8.0.3 to obtain *Pgt* specific and barley specific genes, respectively. The reads that were less than 90% identical for 90% of the read length and mapped to more than 10 positions were selected as unmapped reads. The unmapped reads from all 24 samples were concatenated to generate a single input fastq file. Since the input files contained > 300 M reads, Trinity’s in silico normalization was done to reduce the number of reads for final assembly. The normalized reads were used as the input to generate a de novo transcript assembly. Trinity was run with the parameter ‘--min_kmer_cov 2’ to reduce the total RAM requirement.

The trinity genome-guided RNA-seq and de novo assemblies were combined to obtain a single FASTA file and cleaned using the PASA ‘seqclean’ utility. The PASA assembly pipeline default parameters for single-end reads with two rounds of PASA annotation were used to obtain an updated gene set. A FASTA file containing the PASA updated gene set was generated using the bedtools getfasta utility [87]. The de novo assemblies were generated solely to update the *Pgt* gene models. Any de novo transcripts did not aid in reannotating the *Pgt* gene models were not considered for further analysis.

### Identification of differentially expressed host and pathogen genes

Quantitative expression analysis for barley and *Pgt* were assessed to identify differentially expressed genes (DEGs) between samples inoculated with RMRL virulent and avirulent isolates as well as *Rpg1* virulent and avirulent isolates. The total reads mapped to each gene model for barley and *P. graminis* were normalized to obtain reads per kilobase of exon model per million mapped reads (RPKM) expression values for each sample [88]. The Exact Test in the EdgeR bioconductor package [89] embedded in CLC genomics was used to calculate the fold change and False Discovery Rate (FDR) corrected *p*-value for all comparisons. Genes with fold change > 3 and FDR corrected p-value < 0.05 were considered as differentially expressed genes (DEGs).

The average RPKM expression values of *Pgt* genes across the 24 samples used in this transcriptomics study were used to categorize *Pgt* genes based on gene expression. The *Pgt* genes were categorized into five expression groups: gene expressed as (a) extremely low (< 0–10 RPKM), (b) low (11–50 RPKM), (c) moderate (51–100 RPKM), (d) high (100–500 RPKM) and (e) extremely high (> 500 RPKM).

### Functional annotation and gene enrichment analysis of DEGs

The protein domains of *Pgt* genes were predicted using Hmmscan by searching against all available profile HMM databases (HmmerWeb version 2.30.0 [90];). For the gene enrichment analysis of *Pgt* DEGs, the publicly available gene ontology (GO) term mapping was used for *Pgt* race SCCL genes. For barley, most of the high confidence genes are annotated in the publicly available IBSC RefSeq v1.0 [86]. We supplemented the annotations by performing a local BlastX of the whole set of predicted barley proteins to the reannotated *Arabidopsis* Col-0 genome (Araport11) [91, 92]. The top hits of high confidence barley genes with predicted amino acid homologies greater than 30% and alignment lengths greater than 50% with *Arabidopsis* annotated genes were used to assign *Arabidopsis* gene IDs to the barley genes. The GO term mapping for the best *Arabidopsis* gene hits for the barley DEGs were used for gene enrichment analysis.

In both cases, the GO term enrichment analysis was done in the bioconductor R package TopGO version 2.28.0 [93, 94]. A GO term was considered significantly enriched if more than 5 genes were annotated for that term with classic Fisher p-value less than 0.001 using the Fisher’s exact test performed in the TopGO package. Significantly enriched GO terms were observed only for the differentially expressed barley genes in a comparison between RNAseq libraries from samples inoculated with RMRL virulent versus RMRL avirulent isolates. The enrichment analysis was done to identify significantly enriched GO terms specific to subontology molecular function (MF), biological processes (BP) and cellular component (CC) to provide a better understanding of the molecular activity, biological role and cellular location of the proteins encoded by the DEGs.

### Variant calling using RNAseq data

The quality RNAseq reads were mapped to the *Pgt* race SCCL reference genome sequence in Spliced Transcripts Alignment to a Reference (STAR) software using a two-pass alignment step [80, 95]. The two-pass step utilized the splice junction loci identified in the first mapping to guide the second mapping. The mapped data were sorted, and the PCR duplicate reads were tagged using Picard Mark Duplicates [96]. The SplitNCigarReads tool in GATK was used to split reads into exons and hard-clip overhanging intronic sequence. This command was supplemented with the ReassignOneMappingQuality read filter to convert the alignment quality assigned by STAR to a GATK compatible quality score. Base recalibration was done using the already known variant sites in the *Pgt* race SCCL genome and publicly available in Ensemble Fungi [97]. The variants were called individually for each sample using GATK HaplotypeCaller tools in ERC GVCF mode with the parameters suggested for RNAseq data [80]. The individual variants were combined using GATK GentoypeGVCFs tool to obtain VCF files containing variant calls for all the samples. Variants with genotype qualities greater than 10 and read depth greater than 6 were selected using Vcftools [81]. All the variants, including multiallelic sites present in filtered VCF files, were used as input in the Ensembl Variant Effect Predictor (VEP) tool to identify non-synonymous variants [46]. The multiallelic sites and variants with synonymous mutations were removed from the analysis. Only, the biallelic sites that have predicted non-synonymous mutation that contained genotypic data for more than 50% of the isolates with a minor allele frequency (maf) > 0.01 were selected for the transcriptome-wide association study.

### Transcriptome-wide association study

Transcriptome-wide association analysis was done using the high-quality polymorphic variants identified from RNAseq to identify variants that were significantly associated with virulence on the barley lines containing the RMRL or *Rpg1* stem rust resistance genes. The phenotypic data were generated by transforming the stem rust infection types to quantitative data using the conversion formula provided by [98]. The infection types of HQ1 and Morex were used to identify variants associated with virulence on RMRL and *Rpg1,* respectively. The phenotyping data was combined with the genotypic data containing filtered variants. To correct for the population structure in the isolates, principal component analysis (PCA) was performed in JMP® Genomics v8.0 using the default setting. Three PCA explained more than 25% of the variation in both the RAD-GBS and RNAseq data, thus, three principal components were used in the association mapping (AM) to correct for population structure in the Q (association analysis with correction for the population structure only) and QK models (association analysis with correction for the population structure and familial relatedness). The output from the kinship matrix analysis was used to correct for familial relatedness in the QK model for AM analysis. Along with the Q and QK models, AM was also performed with the naïve model (no correction for population structure and familial relatedness) to identify effectors/suppressors associated with phenotypes on RMRL and *Rpg1* containing barley lines. All the significant variants were manually inspected to eliminate false positives. Variants potentially associated with virulence/avirulence for *Rpg1* and RMRL, but not detected in AM analyses using the Q and/or QK models were also manually inspected to avoid false negative calls for each variant. If a variant had an alternate call, either heterozygous or homozygous for the alternate allele, for more than 75% of virulent isolates (maximum of 2 outlier calls) and homozygous reference call for more than 80% of the avirulent isolates (maximum of 2 outlier calls), then the variant was selected as significantly associated with virulence/avirulence for the specific stem rust resistances mediated by RMRL or *Rpg1*.

### Prediction of candidate secreted effector proteins (CSEPs)

To facilitate the identification of candidate fungal effectors involved in barley R gene specific virulence/avirulence, the annotated *Pgt* genes were searched for effector signatures utilizing bioinformatics analysis. The *Pgt* genes that met the criteria based on; i) the presence of an N-terminal signal peptide; and ii) prediction as an effector using EffectorP software [43, 99, 100] were considered to encode candidate secreted effector proteins (CSEPs). SignalP 4.1 was also used to predict the presence of putative N-terminal secretion signals [101]. The differentially expressed *Pgt* genes, and the genes associated with virulence/suppressors of resistance were annotated as CSEPs based on these prediction criteria.

## Supplementary information

**Additional file 1:** Contains supplementary tables Table S1-S13. Microsoft Excel Workbook containing 13 tables. **Table S1.** Group of *Pgt* isolates based on their virulence on barley lines containing stem rust resistance gene *rpg4/5* and/or *Rpg1*. **Table S2.** Infection type of group1 *Pgt* isolates on barley differential lines. **Table S3.** Infection type of group2 *Pgt* isolates on barley differential lines. **Table S4.** Infection type of group3 *Pgt* isolates on barley differential lines. **Table S5.** Read and mapping statistics of RAD-GBS data of *Pgt* Samples. **Table S6.** RNAseq read mapping statistics for the *Pgt* inoculated Harrington samples. **Table S7.** RNAseq read mapping statistics for the non-inoculated Harrington samples. **Table S8.** Differentially expressed gene in between virulent *rpg4/5* and avirulent *rpg4/5* inoculated sampled and its comparison with the non-inoculated controls. **Table S9.** Gene enrichment analysis of differentially expressed gene in virulent vs avirulent *rpg4/5* inoculated Harrington in R bioconductor package TopGO, **Table S10.** List of gene that are differentially regulated in *Rpg1* virulent isolates compared to *Rpg1* avirulent isolates. **Table S11.** List of gene that are differentially regulated in *rpg4/5* virulent isolates compared to *rpg4/5* avirulent isolates. **Table S12.** Identification of *Pgt* Candidate Secreted Effector Protein (CSEP) and their classification based on level of expression. **Table S13.** Variants associated with *Pgt* virulence on *rpg4/5*.

**Additional file 2.** A FASTA file containing coding sequences of the updated gene models of *Pgt* using the transcript sequences generated from this study in addition to the publicly available gene models of reference genome of *Pgt* race SCCL.

**Additional file 3.** A FASTA file containing amino acid sequences of the updated gene models of *Pgt* using the transcript sequences generated from this study in addition to the publicly available gene models of *Pgt* race SCCL.

## Data Availability

The raw sequence data is deposited to the NCBI database under BioProject PRJNA540228 (GEO: GSE130423) with the sample accession numbers SAMN11525979 - SAMN11526005.

## Abbreviations

CSEP: Candidate Secreted Effector Proteins
DEG: Differentially Expressed Genes
EIS: Effector Induced Susceptibility
ETI: Effector Triggered Immunity
GO: Gene Ontology
INDEL: Insertion/Deletions
MAF: Minor Allele Frequency
MTA: Marker Trait Association
ND: North Dakota
NLR: Nucleotide-binding domain leucine-rich repeat
*Pgt*: *Puccinia graminis* f. sp. *tritici*
PTI: Pathogen Triggered Immunity
RAD-GBS: Restriction Site Associated DNA-Genotyping by Sequencing
RMRL: *rpg4*-mediated resistance locus
RPKM: Reads Per Kilobase of exon model per Million mapped reads
SNP: Single Nucleotide Polymorphism (SNPs)
